# Rapid segmentation of computed tomography angiography images of the aortic valve: the efficacy and clinical value of a deep learning algorithm

**DOI:** 10.3389/fbioe.2024.1285166

**Published:** 2024-05-30

**Authors:** Yu Mao, Guangyu Zhu, Tingting Yang, Ruediger Lange, Timothée Noterdaeme, Chenming Ma, Jian Yang

**Affiliations:** ^1^ Department of Cardiovascular Surgery, Xijing Hospital, Air Force Medical University, Xi’an, China; ^2^ School of Energy and Power Engineering, Xi’an Jiaotong University, Xi’an, Shaanxi, China; ^3^ Department of Cardiovascular Surgery, German Heart Center Munich, Munich, Germany; ^4^ Department of Cardiology, Hospital of Liege University, Liege, Belgium; ^5^ Nanjing Saint Medical Technology Co., Ltd., Nanjing, China

**Keywords:** aortic valve, transcatheter aortic valve replacement, computed tomography angiography, deep learning, automatic segmentation

## Abstract

**Objectives:**

The goal of this study was to explore the reliability and clinical value of fast, accurate automatic segmentation of the aortic root based on a deep learning tool compared with computed tomography angiography.

**Methods:**

A deep learning tool for automatic 3-dimensional aortic root reconstruction, the CVPILOT system (TAVIMercy Data Technology Ltd., Nanjing, China), was trained and tested using computed tomography angiography scans collected from 183 patients undergoing transcatheter aortic valve replacement from January 2021 to December 2022. The quality of the reconstructed models was assessed using validation data sets and evaluated clinically by experts.

**Results:**

The segmentation of the ascending aorta and the left ventricle attained Dice similarity coefficients (DSC) of 0.9806/0.9711 and 0.9603/0.9643 for the training and validation sets, respectively. The leaflets had a DSC of 0.8049/0.7931, and the calcification had a DSC of 0.8814/0.8630. After 6 months of application, the system modeling time was reduced to 19.83 s.

**Conclusion:**

For patients undergoing transcatheter aortic valve replacement, the CVPILOT system facilitates clinical workflow. The reliable evaluation quality of the platform indicates broad clinical application prospects in the future.

## 1 Introduction

Alain Cribier’s completion of the first transcatheter aortic valve replacement (TAVR) procedure in 2002 introduced a new era of treatment for aortic valve (AV) diseases (Cribier, 2002). After more than 20 years of continuous development, the safety and effectiveness of TAVR have been confirmed by a large number of clinical studies. It is now a reliable treatment for patients with AV disease (Leon et al., 2016; Mack et al., 2019; Popma et al., 2019). Recent clinical studies have suggested that preoperative evaluation is a crucial part to guarantee the success of TAVR, which has a significant impact on the procedures and prognosis. The pre-operative evaluation mainly relies on computed tomography angiography (CTA) images, which has been deeply integrated into daily clinical practice. However, CTA images can only provide a two-dimensional (2D) field of view, which not only requires clinicians to have rich clinical experience to build a mental concept of the anatomy derived from 2D CT images displayed on a screen, but also hinder the training of junior doctors and doctor-patient communication (Queirós et al., 2017; Chessa et al., 2022).

With the continuous promotion of interdisciplinary collaboration between medicine and engineering, the advanced techniques based on 3D reconstruction technique provide powerful tools to bridge this gap by intuitively visualizing patient-specific morphology. Segmenting the aortic valve from medical images is the first and most critical step in its 3-dimensional (3D) reconstruction. Traditionally the segmentation procedure mainly relies on manual operations (Maragiannis et al., 2014; De Jaegere et al., 2016; Qian et al., 2017; Haghiashtiani et al., 2020). In addition to requiring specialist knowledge, manual segmentation is still time-consuming and labor-intensive, which cannot meet the clinical demands on a large scale and real-time data processing. Moreover, the quality and reproducibility of manual segmentation are difficult to guarantee, and human errors induced in the manual segmentation stage could result in inaccurate or even wrong clinical decision (Queirós et al., 2017).

With the development of computer science, artificial intelligence (AI) provided a new approach to the automatic segmentation of medical images. As an important branch of AI, deep learning (DL) methods represented by convolutional neural networks (CNNs) have gradually been used in the automatic segmentation of the aortic valve. Among them, Fan et al. proposed the first DL method for aortic valve segmentation which has achieved outstanding performance in segmenting the whole valve structures, helping surgeons with the diagnosis of aortic diseases and planning of TAVR (Fan et al., 2019). With the proposal of novel CNN architectures, Yang et al. compared the comprehensive performance of the four popular 3D CNNs on segmentation quality and efficiency, which demonstrated that 3D Res-UNet is the most appropriate 3D CNN architecture for the automatic segmentation of aortic root under small samples (Yang et al., 2023). The importance of automatic segmentation in the treatment of aortic valve diseases is beyond just time-saving and error reduction. The transformative impact of automatic segmentation technology on clinical practice and patient care could be emphasized in several benefits, such as improved diagnostic accuracy by providing precise 3D reconstructions that facilitate better understanding of patient anatomy, enhanced surgical planning through rapid access to detailed valve and surrounding structures imagery, improved patient communication by offering intuitive images that aid in explaining medical conditions and treatment options, increased training efficiency for medical students and junior doctors by serving as effective educational tools, promotion of interdisciplinary collaboration between medicine and engineering to develop advanced medical tools, and ultimately, the potential for better patient outcomes through personalized medicine and tailored treatment plans. Despite that the recent DL-based studies have shown great potential in streamlining clinical tasks, the reliability and clinical validation performance of DL methods remain to be explored.

Therefore, this study aimed to develop a deep learning (DL) tool for automatic 3D AV reconstruction named CVPILOT system (TAVIMercy Data Technology Ltd., Nanjing, China), whose pipelines includes fully automatic pre-processing, DL-based segmentation, and post-processing. The segmentation performance and clinical performance of CVPILOT system were evaluated on a retrospective cohort with CTA scans.

## 2 Materials and methods

### 2.1 Study population

We retrospectively analyzed the CTA images of 195 patients who underwent TAVR from January 2021 to December 2022 in Xijing Hospital. Twelve patients whose images were of poor quality due to artifacts (n = 9) or to poor image contrast (n = 3) were excluded. Finally, 183 patients were enrolled in the study, and the overall cohort was divided into the training data set (n = 123), the validation data set (n = 31), and the clinical evaluation data set (n = 29). The study protocol was approved by the ethics committee of the hospital (ethics committee approval number: KY20230106-01-KS-01), and all patient data were desensitized.

### 2.2 Definition

Bicuspid aortic valve refers to dysplasia of the aortic valve, which leads to only two leaflets with ≤3 antagonistic borders between leaflets. At present, the most commonly used classification method is the Sievers’ classification (Sievers and Schmidtke, 2007), which is divided into type 0 (no raphe), type 1 (one raphe), and type 2 (2 raphes) according to the number of raphes.

### 2.3 Preoperative CTA image acquisition and analysis

All patients were examined with a dual-source computed tomography (CT) scanner (Definition Flash, Siemens Healthcare, Florsheim, Germany). The scanning parameters included a collimator width of 128 × 0.6 mm. The axial image was reconstructed using the optimal systolic period with a layer thickness of 0.75 mm. Currently, structural cardiologists use the 3mensio system (for pre-TAVR assessment) (Pie Medical Imaging, Maastricht, Netherlands). All patient CT images were in Digital Imaging and Communications in Medicine format. Scan sequences from cohorts were evaluated by two experts. All disputes were resolved by an experienced arbitration expert, and consensus was reached in case of disagreement. The two experts were both structural cardiologists with more than 10 years of professional experience. Segmentation notes include the ascending aorta (AA), the AV, calcification, and the left ventricle (LV).

### 2.4 Data preprocessing and augmentation

All images were interpolated to images with 0.5 mm³ isotropic spacing using trilinear interpolation. Label data were interpolated to the same spacing using the nearest interpolation. After normalizing, voxels with an intensity less than 0 and larger than 1200 were clipped into the thresholds to avoid interference caused by different backgrounds. Non-body regions in the images were cropped to boost training efficiency and the foreground/background ratio.

A TorchIO-based multiple serial data augmentation strategy was then used to ensure the robustness and generalization, specifically, 3-dimensional rotation with ±20° to mimic different orientations of the heart, 3-dimensional isotropic zoom with 0.85–1.15 to mimic different heart sizes, random Gaussian noise and random artifacts to mimic compromised imaging quality.

### 2.5 Training and inference

We use nnU-Net as the principal architecture in our network (Isensee et al., 2021). The whole nnU-Net included several individual U-Nets including the 2D full-resolution U-Net, the 3D full-resolution U-Net, and the low-resolution 3D U-Net combined with a cascade U-Net.

Before training, the nnU-Net scans the whole data set and gives a set of recommended hyperparameters. All weights in the model are initialized, and the model is trained with an Adam optimizer. Each 3D convolutional layer is followed by a LeakyReLu function. The Sigmoid function is used to predict the final segmentation results. During training, the isotropic CT image was cropped into patches with the shape of 256*256*256 as the input of the model. The loss function we used is a combination of Dice loss and focal loss. For objects with a large volume like the AA and the LV, Dice loss can supervise the segmentation well, whereas for objects with a small volume like the AV and calcification, focal loss makes a smoother gradient. After training, a model was compiled using the TensorRT format in order to accelerate inference time and conserve the amount of graphics processing unit memory consumed.

All the experiments in this study were implemented based on Python 3.9 and PyTorch 1.10.1 TensorRT 8.5.0 running in an Ubuntu 20.04 LTS server (Canonical Ltd., London, United Kingdom) equipped with two Intel Xeon Platinum 8375C (INTEL, Santa Clara, CA, United States) and two GeForce RTX 3090 (NVIDIA, Santa Clara, CA, United States) with 24 GB of memory.

### 2.6 Postprocessing and determination of cuspid numbers

In the validation stage, postprocessing was first performed to eliminate scattered segmentation pieces by removing non-largest algorithm to enhance the overall pipeline segmentation performance. After postprocessing, the three mini-batch K-means clustering algorithms (k = 2,3) was performed to evaluate the number of cuspids. After the number of cuspids was calculated, K-means inference was used to obtain a rough re-segmentation, and K-nearest Neighborhood algorithm was used to further determine more precise boundary between two leaflets. Finally, two or three leaflets were named according to their relative and absolute positions. A confusion matrix was drawn to compare the expert label and the machine learning results.

### 2.7 Segmentation quality evaluation

The comprehensive quality evaluation was divided into two parts, including classical segmentation quality evaluation metrics, and clinical expert rating. We selected five classical metrics which were widely used in segmentation quality assessment in this study, including recall, precision, Dice similarity coefficient (DSC), Hausdorff distance (HD) and average symmetrical surface distance (ASSD). In order to explore the clinical performance of the segmentation results, the clinical expert rating was performed with the following criteria (Menzel et al., 2000): (1) grade A: excellent visualization of aortic wall, annulus, leaflet, and calcification (good modeling quality or equivalent); (2) grade B: good visualization of aortic root tissues with small artifacts, but sufficient for diagnosis (slight decline in modeling quality); (3) grade C: the image quality was insufficient to diagnose or may lead to misdiagnosis (poor modeling quality).

### 2.8 Statistical analyses

The Kolmogorov-Smirnov test was used to evaluate the normality of the distribution. The measurement data conforming to normal distribution were represented by the mean ± the standard deviation, whereas the quantitative data not conforming to normal distribution were represented by the median and the quartile range. The result of the classified data was expressed as n (%). Bilateral *p*-values <0.05 were considered statistically significant. All statistical analyses were performed using SPSS version 26.0 (IBM, Armonk, NY, United States).

## 3 Results

### 3.1 Baseline characteristics

**FIGURE 1:**
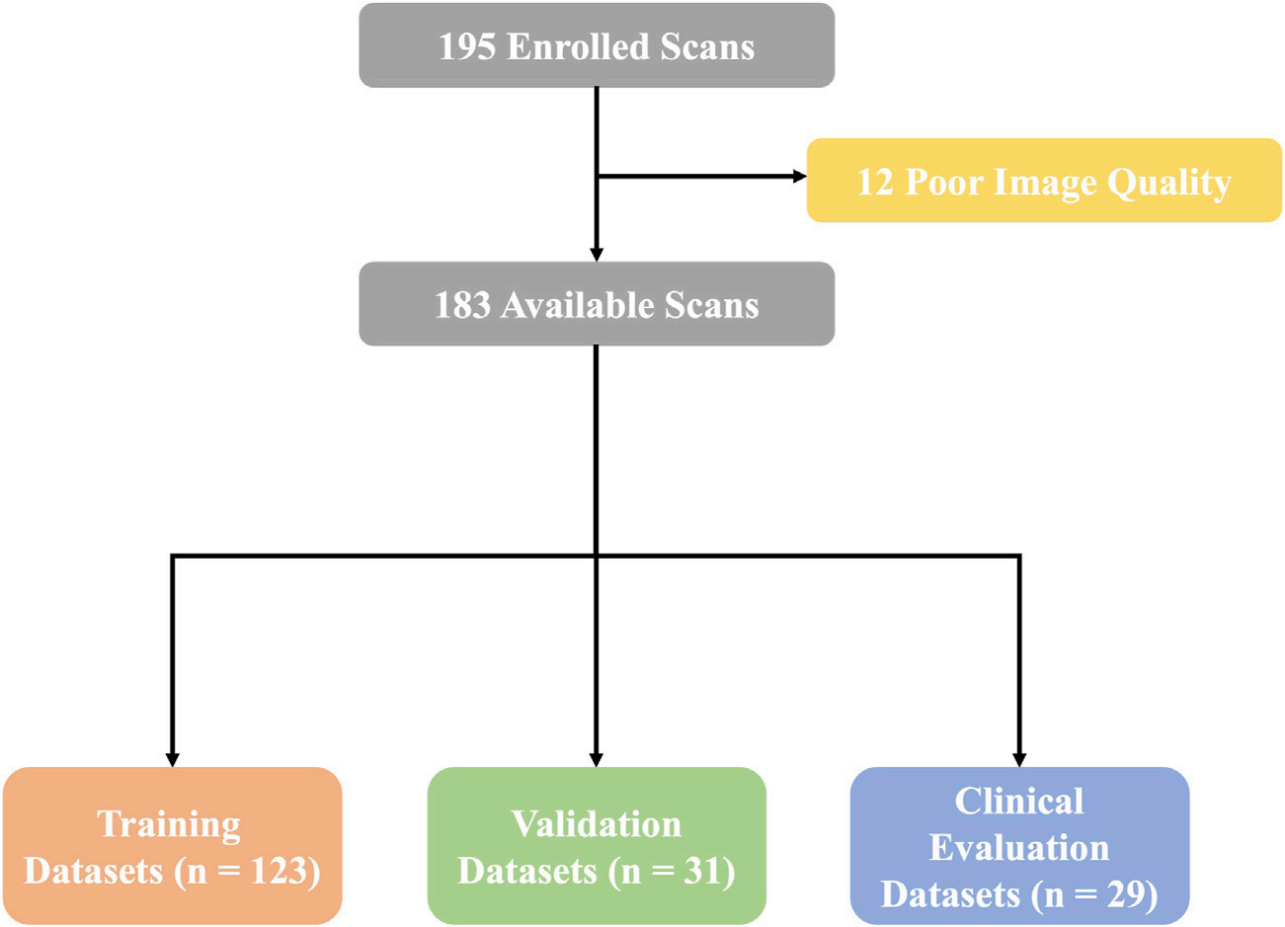
Flow chart. A total of 183 available scans were finally enrolled in this study. Then, the overall data sets were separated to complete training, validation, and clinical evaluations.

A total of 183 retrospective patients subjected to CTA of the aorta were included in this study. The mean age of the patients was 69.3 ± 8.4 years; 107 patients (58.5%) were male and 76 patients (41.5%) were female. Patient cohort characteristics and baseline characteristics of the CTA scans used for training, validation, and clinical evaluation of the data sets are summarized in Table 1. The flow chart of this experimental design is shown in Figure 1.

**TABLE 1:** Baseline characteristics.

Parameters	Training data sets (n = 123)	Validation data sets (n = 31)	Clinical evaluation data sets (n = 29)
Patient characteristics			
Age, y	68.9 ± 8.0	69.7 ± 7.5	69.4 ± 7.2
Male, n (%)	57.7 (71)	54.8 (17)	65.5 (19)
*Diagnosis*			
Aortic stenosis, n (%)	30.9 (38)	42.0 (13)	34.5 (10)
Aortic regurgitation, n (%)	28.5 (35)	29.0 (9)	37.9 (11)
Combined, n (%)	40.6 (50)	29.0 (9)	27.6 (8)
*Leaflet morphology*			
Tricuspid AV, n (%)	71.5 (88)	74.2 (23)	65.5 (19)
Type 0 bicuspid AV, n (%)	6.5 (8)	6.4 (2)	13.8 (4)
Type 1 bicuspid AV, n (%)	21.9 (27)	19.4 (6)	20.7 (6)
Manufacturer information			
Siemens, n (%)	85.4 (105)	90.4 (28)	96.6 (28)
GE (%)	2.4 (3)	3.2 (1)	0
Philip, n (%)	12.2 (15)	6.4 (2)	3.4 (1)

AV, aortic valve.

### 3.2 Model performance

**FIGURE 2:**
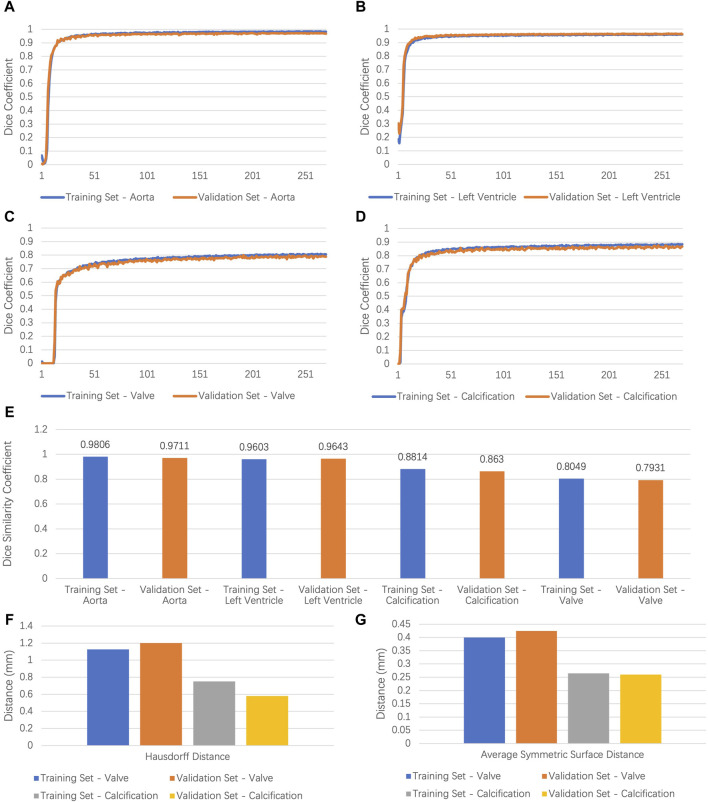
Results of model performance. **(A–D)** Dice coefficient values for the ascending aorta, left ventricle, leaflets, calcification in training, and validation set of the segmentation task. **(E)** Dice similarity coefficient values for the ascending aorta, left ventricle, leaflets, and calcification in training and validation set. **(F,G)** Hausdorff distance and average symmetrical surface distance of leaflets and calcification in training and validation set. AA, ascending aorta; LV, left ventricle.

A total of 123 original CTA images were used for training, and 31 images were used for validation. We deployed nnU-Net 3D full resolution architecture to train our segmentation task. The model was trained by 251 epochs and was converged by about the 200th epoch. Figures 2A–D demonstrate the evolution of the Dice coefficient of the training set and the validation set during model training. We picked the best model for further performance testing after training. As shown in Figure 2E, the segmentation of the AA and the LV attained a DSC of 0.9806/0.9711 and 0.9603/0.9643 for the training and validation set, respectively. The leaflets had the DSC of 0.8049/0.7931, the precision of 0.7882/0.7656, and a recall of 0.8359/0.8302 for the training and validation set, respectively. The calcification area had the DSC of 0.8814/0.8630, the precision of 0.8829/0.8568, and the recall of 0.8568/0.8750 for the training and validations sets, respectively.

For calcification and leaflets, both DSC and morphology consistency matter. To evaluate shape consistency, we calculated the HD and the ASSD between the prediction and the ground truth. As shown in Figures 2F,G, 95% of the HD for calcification was 0.7520/0.5797 mm for the training and validation set, respectively. The ASSD for calcification was 0.2648/0.2602 for the training and validation set, respectively. Furthermore, 95% of the HD of the leaflets was 1.1272/1.201 mm for the training and validation set, respectively. The ASSD of the leaflets was 0.3999/0.4241 mm for the training and the validation set, respectively. Generally, the segmentation performance for calcification was better than that for the leaflets, which may be due to the larger contrast for calcification and the movement artifacts of the leaflets.

Additionally, we timed the modeling for manual and automatic approaches. The average modeling time for radiologists using Mimics Version 21.0 (Leuven, Belgium) was 31,767.3 s. However, the CVPILOT pipeline only takes an average of 19.83 s and a maximum of 59.39 s, depending on the range and resolution of the CTA scans.

### 3.3 Determination of the cuspid numbers and re-segmentation

**FIGURE 3:**
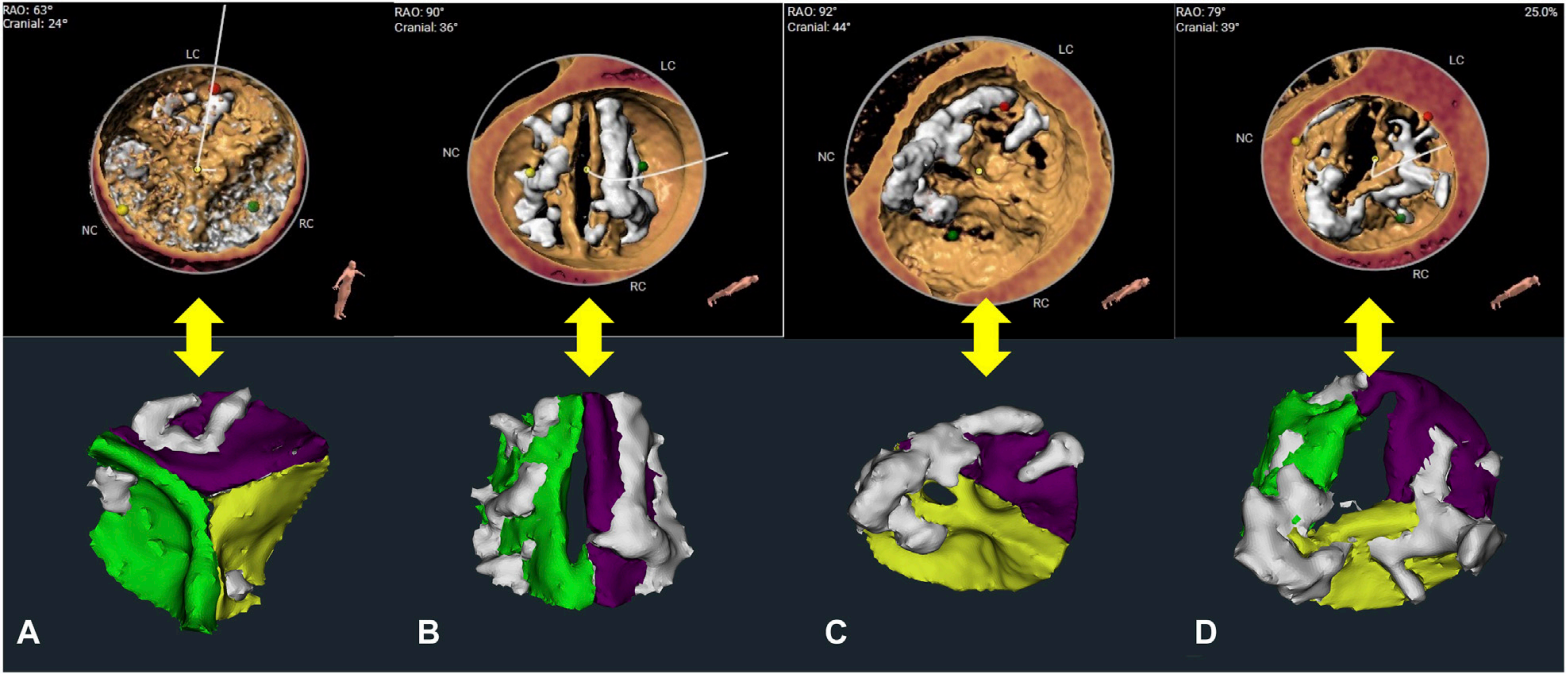
Leaflet number determination and re-segmentation. **(A–C)** show the structures of the tricuspid aortic valve (TAV), type 0 bicuspid aortic valve (BAV), type 1 BAV, reconstructed using the 3mensio system and the CVPILOT system, respectively. **(D)** shows the error example (mis-identifying type 1 BAV as type 0 BAV) reconstructed using the CVPILOT system.

The confusion matrix demonstrated that the overall reconstruction accuracy was 0.923. The proportion of bicuspid aortic valve (BAV) was 27.9% (43/154), and 72.1% (111/154) for tricuspid aortic valve (TAV) cases. The reconstruction accuracy of BAV and TAV cases was 0.904 and 0.947, respectively. Most error cases were mis-identifying type 1 BAV cases as type 0 BAV cases (Figure 3). This result illustrates the clinical similarity of these two kinds of patients.

### 3.4 Clinical quantitative evaluation of segmentation

**FIGURE 4:**
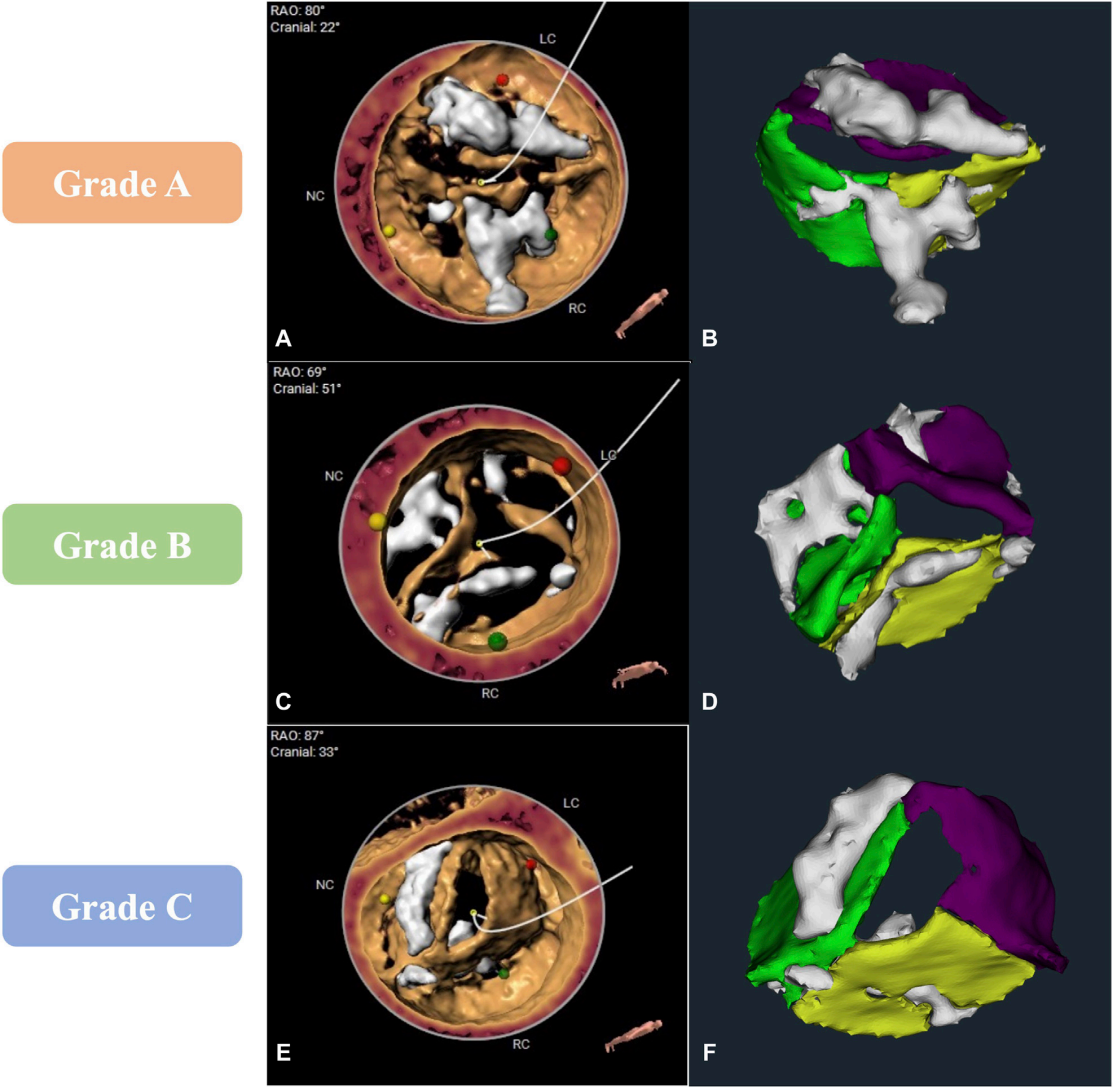
Comparison of computed tomography angiography image analysis results between 3mensio and the CVPILOT system. **(A,B)**, **(C,D)**, and **(E,F)** represent images reconstructed by the CVPILOT system that reached grade A, B, and C, respectively.

In our clinical evaluation stage, we evaluated the model in terms of whether its segmentation results met the requirement of high-precision modeling. Among 29 cases in the clinical evaluation stage, 48.3% (n = 14) cases achieved grade A, 44.8% (n = 13) cases achieved grade B, and 6.9% (n = 2) cases were Grade C. Compared to the grade A cases with clear leaflet boundaries, correct calcification distribution, and precise AV areas (Figures 4A,B), despite that the grade B cases (Figures 4C,D) showed that the major contour and shape from automatic segmentation match 3mensio volume rendering and relatively good calcification segmentation, it showed a common problem on segmentation of the leaflet commissure region, which the segmented boundary may shift for about 3 mm (Figure 4D). In grade C cases, the biggest problem was that the leaflets showed over- or under-segmentation, which may be caused by motion artifacts (Figures 4E,F).

## 4 Discussion

In this study, we proposed an algorithm pipeline named CVPILOT that integrates deep learning and machine learning with segment aortic root structures in CTA scans. This system can segment aortic root structure, recognize different leaflet structures, and give a high-precision adaptive leaflet segmentation result.

In the past, imaging data were obtained by 2D imaging methods such as CTA, echocardiography, and magnetic resonance, and the lesion area was accurately identified by image segmentation, so that surgeons could achieve accurate diagnoses, preoperative planning, and postoperative monitoring. However, 2-dimensional images are usually characterized by complex tissue textures, blurred boundary regions, and low contrast, which greatly limits the effects and application scenarios of traditional image segmentation methods (such as the threshold method and the regional growth method) (Menzel et al., 2000; Blanke et al., 2019).

With the rapid development of AI, DL has shown obvious advantages in medical image segmentation tasks (Hahn et al., 2021). The results can better demonstrate aortic root structures compared to the traditional volume rendering method and the manual label approach. DL segmentation is based on nnU-Net, which is the state-of-the-art model currently used in medical segmentation.

Building on the strengths of the nnU-Net architecture, our proposed tool, CVPILOT, introduces several innovative features that set it apart from existing models. Firstly, CVPILOT incorporates a novel attention mechanism that enhances the network’s ability to focus on critical areas of the aortic valve, such as the leaflets and calcifications, leading to more accurate segmentation results. This attention mechanism is particularly beneficial in distinguishing between bicuspid (BAV) and tricuspid (TAV) aortic valves, which is a common challenge in automatic segmentation.

Secondly, CVPILOT utilizes a multi-scale 3D convolutional neural network that processes images at various resolutions, allowing for a more nuanced understanding of the aortic root’s complex geometry. This multi-scale approach improves the segmentation of small and intricate structures, such as the valve cusps, which are essential for precise TAVR planning.

Additionally, our tool employs an advanced data augmentation strategy that simulates a wide range of imaging conditions, including different patient positions, heart rates, and imaging artifacts. This strategy not only enhances the model’s robustness but also ensures that it can generalize well across diverse patient populations and imaging protocols.

In terms of clinical relevance, CVPILOT’s segmentation results are directly actionable, providing surgeons with detailed insights into the aortic valve’s morphology and function. The tool’s output includes not only the segmented images but also quantitative measurements of the valve area, annulus size, and leaflet mobility, which are critical for determining the appropriate TAVR strategy.

Our quantitative evaluations, which include the calculation of Dice Similarity Coefficient (DSC), Hausdorff Distance (HD), and Average Symmetrical Surface Distance (ASSD), demonstrate that CVPILOT achieves high segmentation accuracy, comparable to or surpassing that of manual segmentation by experienced clinicians. Moreover, clinical evaluations have shown that the tool’s segmentations are highly correlated with expert assessments, indicating its potential to serve as a reliable tool in the preoperative evaluation process.

The architecture exhibits great generalizability in fitting, regardless of the presence of aortic stenosis or regurgitation in patients with BAV or TAV structures. We did both quantitative evaluations, which included calculating DSC, HD, and ASSD, and clinical evaluations to test whether automatic segmentation had the ability to assist with diagnosis and pre-TAVI modeling. This study makes fast modeling and precise visualization a reality, whereby these methods will play more important roles in preprocedural modeling and in pre-TAVR modeling going forward.

Previous studies in aortic root structure focused mainly on landmarks, detection of key points, and measurements in patients with TAV structures. There are a few studies on BAV anatomy. Queiros et al. reported an aortic annulus sizing algorithm based on automatic segmentation of aortic root structures (Queirós et al., 2017). They implemented an intensity and shape-based strategy to delineate the AV border. This method relies on a certain topology and may not work well on patients with BAV. Furthermore, there are some studies on aortic root modeling and segmentation using DL or atlas-based segmentation (Gao et al., 2016; Baskaran et al., 2020). However, AV as a key structure was not included. Additionally, Aoyama et al. utilized a cascade DL model to segment TAV structures (Aoyama et al., 2022). However, the segmentation result did not include the LV and calcification. So, the relative position information and calcification distribution may be missed. These studies produced satisfactory results in solving problems and giving answers based on specific aortic root structures. However, the different topology of AV makes it difficult to design an adaptive algorithm.

Our study, to the best of our knowledge, reports a solution that handles both BAV and TAV structures together. So, the adaptive algorithm with a high accuracy of predicting leaflet structures and segmentation is an elegant solution for fast and precise AV modeling and benefits patients suffering from AV diseases. For 2 cases with Grade C, the potential reasons might be due to factors such as imaging artifacts, patient-specific anatomical variations, or limitations in the deep learning model’s ability to generalize from the training data to the 2 cases.

The study has the following limitations. First, the study did not include CTA data on atypical aortic root structures (such as quadricuspid AV) and on patients who had previous coronary artery bypass grafting or AV replacement/repair. These influences may interfere with the DL algorithm and lead to potential errors in the analysis of the anatomy of the aortic root. Furthermore, our two experts have a similar work background. Finally, because the study is a single-center retrospective study, it needs to be verified with more diverse data from more centers.

## 5 Conclusion

Overall, we used a DL algorithm to construct a CVPILOT system to achieve rapid automatic 3D reconstruction and segementation of aortic roots. Compared with manual reconstruction, the system has the advantages of accuracy, speed, and high repeatability. Although the system has not been ready to completely replace manual operations in the analysis of CTA images, it has great potential to improve the efficiency of preoperative evaluation, helping surgeons determine more appropriate procedural strategy.

## Data Availability

The original contributions presented in the study are included in the article/Supplementary Material, further inquiries can be directed to the corresponding author.
